# Lumbar spine coronal balance parameters as a predictor of rehabilitation management outcomes in patients with radiculopathy due to lumbar disc herniation: A multicenter prospective case series study

**DOI:** 10.1016/j.heliyon.2024.e40613

**Published:** 2024-11-23

**Authors:** Yaser AH. Aljallad, Ibrahim M. Moustafa, Mohamed Badr, Nouran Hamza, Paul A. Oakley, Deed E. Harrison

**Affiliations:** a Department of Physiotherapy, College of Health Sciences, University of Sharjah, Sharjah, 27272, United Arab Emirates; b Neuromusculoskeletal Rehabilitation Research Group, RIMHS–Research Institute of Medical and Health Sciences, University of Sharjah, Sharjah, 27272, United Arab Emirates; c Department of Physical Therapy for Neuromuscular Disorders, Al Hayah University, Egypt; d Department of Biostatistics, Mars-Global, London, UK; e Independent Researcher, Newmarket, ON, L3Y 8Y8, Canada; f Kinesiology and Health Science, York University, Toronto, ON, M3J 1P3, Canada; g CBP Nonprofit (a spine research foundation), Eagle, ID, USA

**Keywords:** Low back pain, Lumbar radiculopathy, Disc herniation, Spine radiography, Coronal balance, Case series

## Abstract

This prospective consecutive case series was conducted in 5 physiotherapy clinics in the UAE from January 2021–March 2023 to assess coronal lumbar spine radiographic parameters as a predictor of conservative therapy outcomes in patients suffering from low back and leg pain due to lumbar herniated nucleus pulposus (HNP). Ninety patients (mean age 44 yrs., 54 % male) with lumbar HNP underwent conservative therapy. All participants received lumbar spine MRI and radiography to assess spine alignment. Interventions included specific exercises, diathermy, traction, education, a home-based exercise program, and medications. Detailed demographic data was collected. Follow-up was 6-months after discharge. A successful outcome was based on a minimum of the following four outcomes: (1) reduction of radicular or leg pain by 17.5 points (0–100 NRS); (2) fatigue reduction by 7.5 points; (3) distress reduction by 5 points; and (4) interference reduction by 9.5 points. At 6-month follow-up, it was found that patient age, education, and radiographic lumbosacral angle measures significantly affected the odds of a successful outcome. Increasing age by 1-year significantly decreased the odds of success of improving pain (OR = 0.85, p = 0.016), fatigue (OR = 0.85, p = 0.016) and interference scores (OR = 0.89, p = 0.042) by 15 %, 15 % and 11 %, respectively. Lower education significantly increased the odds of success for improving pain, fatigue, and interference by 26.18, 26.18, and 7.5 (p = 0.006, = 0.006, and = 0.029, respectively). Increasing (worsening) the radiographic lumbosacral angle by each degree significantly reduced the odds of success for improving pain, fatigue, distress, and interference by 3.52, 3.52, 27.99 and 2.55, respectively (p < 0.001, <0.001, = 0.003, = 0.001). Our findings indicate that younger age, less education, and better coronal radiological lumbar spine alignment all had a substantial impact on the likelihood of success on 6-month outcomes in patients suffering from chronic lower back pain and radiculopathy due to HNP.

## 1 Introduction

Lumbosacral radiculopathy associated with a disk herniation or herniation of the nucleus pulposus (HNP) is a common clinical problem strongly associated with delayed recovery, persistent disability and increased healthcare utilization and costs [1]. The reported prevalence of radiating leg pain varies from 1 % to approximately 40 % and has a large financial burden increasing healthcare and economic costs of general low back pain by approximately two thirds [2]. Though debated, in general, no relevant gender difference in the occurrence of HNP and radiculopathy has been identified [3]. Despite the high prevalence of this condition, its conservative treatment remains challenging for spine specialists and 25–50 % of patients will continue to have symptoms for a year or longer [[2], [3], [4], [5]]. Recent systematic reviews reported a lack of clearly effective conservative treatments for lumbar radicular pain, particularly for long-term management [6,7]. Most important, the literature provides scant information about the relevant biopsychosocial patient variables that may be the explanatory reasons for those patients who will have suboptimal outcomes at long-term assessment following participation in rehabilitation programs [8]. The prognosis for conservative treatment needs to be determined in a timely and efficient manner to allow health professionals to decide whether the lumbar HNP patient requires surgery or continues non-surgical management [9].

Treatment of HNP consists of both non-surgical and surgical procedures. Many patients prefer conservative treatment over surgery because it carries a lower risk of complications and lower cost [10], however, some investigations have identified that after failed conservative intervention for a course of 6-weeks, further care is of minimal value as it increases costs and has limited effectiveness. Surgery is recommended when HNP is severe, lasts longer than six weeks, or fails to improve with conservative treatment [11,12]. However, investigations looking at the optimum duration of conservative care and the most effective types of treatment for each specific patient subgroup are still lacking [13]. Thus, the need for investigations identifying patient-specific parameters that predict the odds of successful conservative treatment and the optimum types of interventions for lumbar HNP are crucial. Specific predictors can help target and improve treatment options for patients as well as offer the potential to enhance clinical care and improve prognosis for long-term care plans. Some predictors such as magnetic resonance imaging (MRI) classification, pain intensity, duration of complaints, response to the straight-leg raising test (SLRT), and grade of motor power have been reported previously [11,12]. While the type of disc herniation (bulge, protrusion, or extrusion) is poorly correlated with clinical signs and symptoms [14], specific features such as axial HNP area, percentage of canal compromise, cephalad or caudal HNP migration, and type of signal intensity have been found to be predictive of patients who need and respond favorably to microscopic lumbar discectomy [12].

Despite the fact that many studies have described the important role of abnormal spine/posture alignment, which is considered by some to be an important etiological factor for low back pain and many other pathological changes [15,16], there is little agreement as to what constitutes an 'acceptable' coronal lumbo-pelvic spinal alignment and/or which specific spine measurement variables should be used to define and predict patient outcomes in the majority of cases. It is important to note that the literature on spinal alignment has established that a better spine alignment including coronal and sagittal balance is essential for optimal biomechanical function [[17], [18], [19], [20]] and many clinical trials [[21], [22], [23], [24]] have demonstrated that corrections in patient posture and sagittal plane curvatures have resulted in both short and long-term relief of pain and neurological symptoms in chronic (non-acute antalgic) conditions.

However, reviewing the literature on this topic suggests that most rehabilitation programs do not typically consider spine alignment variables, in particular the coronal alignment parameters. Certainly, achieving spinal sagittal balance has emerged as one of the most important clinical outcomes and has been shown to have a significant effect on patient-related outcomes [[21], [22], [23], [24]]. More recently, imbalance in the coronal plane has been shown to negatively affect patient satisfaction and is associated with elevated pain, loss of function, and decreased quality of life [[25], [26], [27], [28]]. Specifically, the fractional lumbo-sacral curve (FLSC), which is the coronal curvature from L4-S1 and is generally in the opposite direction relative to the primary Cobb angle curvature in the lumbar spine, has been detailed and found to be a predictor of poor patient outcomes in conservative and surgical interventions in adult patient populations suffering from scoliotic deformities [[29], [30], [31], [32]].

However, we could not locate any investigations looking at the FLSC in chronic low back pain patients with radiculopathy due to disc herniations. There seems to be a lack of information on the correlation between chronic disc extrusion size and clinical manifestations of radiculopathy and the exact type or nature of coronal imbalance of the lumbar spine. Thus, it may be relevant to identify if the FLSC is a predictor of patient coronal imbalance resulting from chronic disc herniation shape, location, and magnitude to identify if the FLSC is related to conservative-based interventions and outcomes for this unique population. Accordingly, our study sought to investigate the coronal lumbar spinal alignment as predictor of conservative treatment outcomes in patients with chronic low back pain and primary radiculopathy due to HNP using a multi-center conservative care prospective consecutive case series observational trial with a minimum follow-up of 6-months. We hypothesize that the size of the FLSC and other AP lumbar radiographic coronal measurements would predict treatment outcomes in patients having low back pain and a primary complaint of leg radicular pain due to HNP.

## 2 Materials and methods

### 2.1 Study design and population

This is multicenter, prospective consecutive case series study conducted across 5 physiotherapy clinics in the UAE and Egypt from January 2021 to March 2023 to assess the coronal lumbar radiographic spinal parameters as potential predictors of conservative therapy outcomes in HNP patients suffering from lower back pain and radiculopathy where the patients primary complaint was leg pain. We followed the PROCESS guidelines (http://www.processguideline.com/) for the reporting of case series designs. The protocol of the study was approved by both the Research Ethics Committee at the University of Sharjah [REC-21-03-11-S] and the Research Ethical Committee at Cairo University [P.T.REC/012/002563]. All participants completed written informed consent. Patients with lumbar HNP undergoing first time conservative treatment were included in this prospective consecutive case series. Interventions included the following methods: back rest, a multi-modal physical therapy program consisting of specific low back exercises, diathermy, and spinal traction. Additionally, a home-based exercise program and education was included. Lastly, pharmaceutical prescriptions were provided including nonsteroidal anti-inflammatory drugs (NSAIDs), analgesics, muscle relaxants, and orally administered narcotics.

### 2.2 Exclusion criteria

Exclusion criteria included patients with cauda equina syndrome, pregnancy, who had received spine surgery around the lumbar region, and had significant lumbar deformity. Additionally, patients with an anatomical leg length discrepancy (LLD) greater than 12mm were excluded from our study population due to its association with disc herniation, radiculopathy, and lumbar spine displacements; LLD was verified both by radiography and clinical examination by an Orthopedic surgeon [[33], [34], [35], [36]].

#### 2.2.1 Inclusion criteria lumbar disc herniation

Ninety patients met the criteria and agreed to participate in this prospective, multi-center, consecutive case series investigation. All lumbar HNP with unilateral or bilateral radicular pain and patients aged between 18 and 65 years old were considered eligible including patients with more than 1-level of disc herniation. Demographic data such as age, sex, educational level, marital status, body mass index (BMI), and smoking status were collected. Clinical follow-up was scheduled for the sixth month after discharge from active conservative care treatment.

All the patients underwent a standardized neurologic examination, including sensorimotor testing: 1) Motor testing included evaluation in hip flexion, knee extension, ankle dorsiflexion, hip abduction, and ankle plantar flexion and was assessed with use of a standard ordinal scale (0–5). Only patients with normal or mild weakness, grade 4/5 or greater were included in this study. 2) Sensory testing included pinprick testing at the mid anterior thigh for assessment of the L2 dermatome, the medial aspect of the knee (L3), the medial aspect of the ankle (L4), the dorsal aspect of the great toe (L5), and the lateral border of the foot (S1). Sensation was graded on an ordinal scale (0–2), with “2” representing normal sensation, “1” representing some deficit, and “0” representing absent sensation. Only patients with normal sensation or with a deficit grade of 1 were included.

**Fig. 1:**
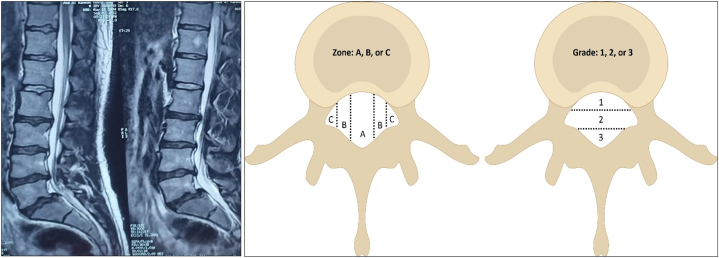
The Michigan State University (MSU) classification system uses a combination of size and location of disc herniation. For example, the location of disc herniation level and location is classified as L2/L3 (A, B, or C) based on its location in these areas medial to lateral. Then the size of the herniation is graded from 1 to 3 where 1 is smaller and 3 is the largest in size; L2/L3 A2 for example. Adapted from Mysliwiec LW et al. [37].

Lumbar HNP was defined as disc herniation that occurred at any mid-lower lumbar levels (L2-S1) and was diagnosed using magnetic resonance imaging. All types of HNP (central, paramedian or foraminal) and all HNP magnitudes (bulge, protrusion, or extrusion) were included. The Michigan State University (MSU) classification for lumbar disc herniation was used as it is a simple and reliable method to objectively measure herniated lumbar discs [37]. The MSU offers a simple classification of disc herniation size as 1, 2, or 3 and provides a location as either A, B, or C; the MSU has high inter-examiner reliability of 98 % [37]. Fig. 1 shows an example of a patient with HNP included in our investigation (note that axial images were assessed as well for all patients) and the MSU classification system is adjacent to the MRI image.

### 2.3 Outcome variables

The success or failure of treatment at 6-months following the completion of active therapies was the primary outcome of interest herein. Patient success or failure was defined using validated thresholds of improvement across the four domains of the patient centered outcome questionnaire (PCOQ).

#### 2.3.1 The patient centered outcome questionnaire (PCOQ)

All patients were administered and completed the PCOQ [38]. The PCOQ assesses the patient's perspectives regarding their presentation, treatment goals, and expectations on four primary domains (pain, fatigue, distress, and interference) and attempts to quantify how each of these four domains has impacted the patient's health and wellbeing and how treatment will or has affected these [39]. In each domain, the patient rates their level of pain, fatigue, distress, and interference of daily activities using a 101-point (0–100) numerical rating scale (NRS) for their typical, hopeful, and wanted level on the scale [39]. The patient is then asked to identify what they expect following their physical therapy intervention and how important they consider an improvement in each of these domains [39].

The following four end points, herein, were chosen based on prior investigations that have established the reliability and validity of these criteria: diminished radicular or leg pain intensity which was the primary patient complaint in our population, diminished fatigue, diminished distress, and diminished interference [39]. A successful outcome of treatment was based on any or all of the four domain outcomes being improved at cessation of care using the minimum cutoff point improvements as established by Brown and colleagues [39] and reduction of on each domain was either maintained or showed continued improvement at 6-month follow-up. In contrast, treatment failure was defined as when the improvements of each of the domains was below the minimal important cutoff values. The optimal cutoff points for success on the 101-point NRS for each of the 4 domains were as follows: (1) improvement in leg pain of at least 17.5 points; (2) improvement in fatigue by at least 7.5 points; (3) improvement in distress of at least 5 points; and (4) improvement in interference of at least 9.5 points. In contrast, failure was defined at 6-month follow-up by any one of the following: (1) an increase in pain or improvement less than 17.5 points; (2) greater fatigue or improvement less than 7.5 points; (3) improvement of distress less than 5 points or an increase; and finally (4) less than a 9.5 point improvement of inference with daily activities [39].

### 2.4 Radiological explanatory variables

**Fig. 2:**
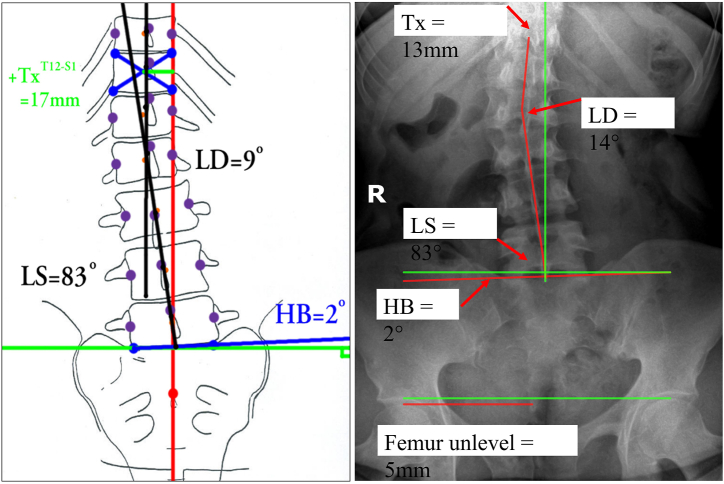
Spine radiographs were analyzed for: 1) a lateral translation of T12 compared to S2 tubercle (TxT12-S1), 2) a mid-lumbar angle (LD), 3) the sacral base to horizontal (HB), and 4) lateral bending of the lower lumbar vertebra compared with the sacral base (LS). This method has excellent reliability [44].

The AP lumbo-pelvic radiographs were analyzed with a modified Risser-Ferguson method, which includes a lateral translation distance of T12 compared with the S2 tubercle (Tx), an angle of coronal bending at mid-lumbar spine (LD), an angle of the sacral base tilt angle relative to horizontal (HB), and an angle of lateral bending of the lower lumbar vertebra compared with the sacral base (LS); note the LS angle here is similar in concept to the FLSC in lumbar scoliotic deformity analysis. This AP radiographic method used herein has been reported to have inter-class and intra-class correlation coefficients in the high ranges with low standard errors of measurement (SEM < 1.5° for angles and SEM < 2 mm for distances) [40]. All the radiographic parameters were measured by a radiologist with 15 years' experience using the MedDream DICOM viewer's radiology measurement tools. Fig. 2 demonstrates this method of AP lumbar radiographic assessment. These measurements are described in further detail here.

• T12-S1 centroid horizontal displacement for trunk lateral translation is measured using a vertical line drawn up from mid-S1 or S2 tubercle. The amount of trunk shift (lateral translation) is measured as the displacement of the centroid (Risser-Ferguson method) of T12 vertebra from this vertical line in millimeters (Tx). Fig. 2.
• Sacral un-leveling is measured relative to a line drawn across the sacral base and compared with a true horizontal line (HB angle). Fig. 2.
• Lumbo Sacral angle is the lower lumbar vertebra centroid line relative to the sacral base line and is the LS angle; this is similar to the FLSC in lumbar scoliotic deformity analysis. Fig. 2.
• Lumbo-dorsal angle or mid lumbar scoliosis angle is similar in concept to a Cobb angle but it uses all the vertebrae to create the angle and thus is more representative of actual lumbar coronal bending alignment. Fig. 2.

### 2.5 Conservative therapy

Our study included a multi-modal, comprehensive interventional treatment program that was customized to the individual designed to improve both pain and functional ability. To treat chronic low back pain and lumbar radiculopathy it is best to address pain, activity limitations, and improve overall function across a persons daily life [[41], [42], [43], [44], [45], [46], [47]].

#### 2.5.1 Protocol of treatment flow

Our study used only treatment facilities that utilized a similar treatment phiolosophy and approach to care. The treatment protocol was guided by a general flow-chart outlining the progression of therapy as follows where each patient received the following interventions: 1) Initially, over a span of four weeks, all patients received treatments such as transcutaneous electrical nerve stimulation (TENS), electromagnetic short wave diathermy (SWD), and mechanical traction; in addition to general exercises aimed at improving muscle strength and mobility. 2) Subsequently, in the following four weeks, the focus shifted to lumbo-pelvic stability exercises aimed at activating deep core muscles. 3) During supervised sessions in weeks nine and ten, patients advanced to more challenging lumbo-pelvic stability exercises using a physio-ball. 4) Finally, from the tenth week onward, patients transitioned to standing positions, engaging in functional movement exercises to enhance balance and coordination. This gradual approach ensured comprehensive enhancement of core strength and stability, effectively addressing the complexities associated with managing chronic lumbar radiculopathy. To ensure fidelity of the treatment protocol, all therapists participating in this study were qualified professionals with a minimum of 10 years of experience in physiotherapy. The intervention was limited to only five therapists across the participating hospitals, ensuring uniformity and consistency in the administration of treatments.

#### 2.5.2 Patient education

Patient Education: education forms the cornerstone of the treatment. The focus is on individualized sessions that delve into the diverse factors contributing to pain, self-management techniques, and the anticipated progression of the condition. Patients were guided on sustaining activity, pacing strategies, and effective methods for safeguarding the back and we focused on six general categories of education:

Understanding Pain: Patients received comprehensive information about the multifaceted nature of pain, including the physiological, psychological, and social factors that contribute to their experience of pain. This understanding helps to reduce fear and anxiety associated with it.

Self-Management Techniques: Patients were equipped with strategies for self-management, including exercises and stretches specifically designed to alleviate pain and improve flexibility and strength. Instruction on proper body mechanics and posture was provided to minimize strain on the lumbar spine during daily activities.

Activity and Pacing: Patients were educated on the importance of maintaining regular physical activity and guided on how to pace their activities to prevent overexertion. Techniques for breaking tasks into manageable steps and taking regular breaks were emphasized to balance activity and rest effectively.

Protecting the Back: Instruction on safe lifting techniques, ergonomic adjustments for workstations, and proper sleeping positions were provided to protect the back from further injury. Patients learned how to engage their core muscles during activities to support the spine.

Coping Strategies: Patients were taught cognitive-behavioral techniques to manage pain-related stress and anxiety. This included relaxation exercises, mindfulness practices, and positive thinking strategies to cope with chronic pain.

Progression and Expectations: Education covered the expected progression of the condition, helping patients set realistic goals and expectations. Information on when to seek further medical advice and how to monitor their condition was included to ensure they remained proactive in their treatment.

#### 2.5.3 Medication administration

All patients received a regimen consisting of 300 mg of gabapentin along with 200 mg of nonsteroidal anti-inflammatory drug (NSAID), Celecoxib, administered twice daily for a period of 6 weeks. Additionally, oral muscle relaxants were administered for a duration ranging from 2 to 3 weeks. A subset of patients (7 patients) were prescribed a short-term course of opioid therapy lasting two weeks. After the initial 6-week period, medication was discontinued for all patients for a period of 4 weeks. During the follow-up supervised sessions, a selective approach was adopted for prescribing NSAIDs. Only patients experiencing persistent pain, approximately totaling 50 individuals, were provided with NSAIDs as needed. This stepwise medication strategy allowed for the adjustment of treatment based on patient response and the evolving nature of their symptoms, ensuring personalized and effective pain management. Overall, the stepwise medication management approach prioritized individualized care, flexibility, and optimization of pain control while minimizing the risks associated with prolonged medication use. Lastly, despite being recognized as a novel therapeutic option for neuropathic pain, neurotrophic drugs were not utilized in this particular study.

#### 2.5.4 Physical treatments TENS, diathermy, and traction

A series of physical treatments were integrated to address each patient's condition. All patients received transcutaneous electrical nerve stimulation (TENS). TENS was applied for 20 minutes on the lower back area, operating at specific parameters: a frequency of 100 Hz, an amplitude of 15 mA, and a duration of 100 milliseconds. Shortwave diathermy (SWD) which has proven with exercise to be effective in reducing pain in patients with chronic low back pain [*41*]. SWD was utilized to induce thermal effects beneficial for chronic cases. This treatment aims to trigger vasodilatation, elevate pain threshold, reduce muscle spasm, accelerate cellular metabolism, and enhance soft tissue extensibility [*42*].

Mechanical lumbar traction was administered in the *prone* position, utilizing intermittent traction for 15 minutes. There is substantial evidence to support the use of prone lumbar traction for patients with chronic low back pain and lumbosacral nerve root involvement [43,44]. While there are other studies that support the efficacy of supine traction, the decision to select prone lumbar traction was the standardization of protocols applied at our 5 clinical settings involved. The traction involved intervals of 30-s holds and 10-s rests. It commenced at 25 % of the patient's body weight and was increased gradually based on individual tolerance, up to a maximum of 50 % of the total body weight [*43*].

#### 2.5.5 Focused exercise regimen

A patient focused exercise regimen was administered. The exercise program involved two primary components: a general exercise program and a lumbar spine stabilization program. First, the general exercise program: this program encompassed limb and spine stretching exercises and strengthening of abdominal and lumbar muscles, tailored to each patient's specific needs. Second, the lumbar spine stabilization exercise (LSSE) program: the LSSE progressive regimen targeted deep core and superficial muscles, emphasizing a neutral spinal position and integration into daily activities. The LSSE program emphasizes the integration of core muscle engagement into daily activities to promote a neutral spine position and enhance overall stability. For instance, engaging the transversus abdominis (TA) while walking helps stabilize the pelvis and lower back, promoting better posture and reducing the risk of back pain [45].

Patients were guided through exercises focusing on lumbar multifidus and transverse abdominus activation, followed by static and dynamic stabilization exercises. Moreover, before lifting objects, it is crucial to engage the TA by performing the abdominal drawing-in maneuver or abdominal bracing. This action helps stabilize the spine and prevent injury [46]. Ensuring proper lifting techniques with a focus on core engagement is essential for lumbar health [46]. These exercises progressively evolved and were integrated into daily living activities and work routines. Both exercise protocols, the general exercise and LSSE programs, spanned a 4-week duration and were conducted three times a week. Sessions lasted 45 minutes, after the first month the patients received supervised sessions once a week [47].

### 2.6 Data analysis

We used counts and percentages to describe categorical data. In order to test normality of the variables we used the Shapiro-walk. Next numeric data was presented as median and interquartile ranges (IQR) for the variables of age, exploratory radiological data, and the scores of the 4 domains of the PCOG as these were not normally distributed. Multiple logistic regression models were used to assess the predictors of the success of each of the 4 outcomes as well as the 4 outcomes combined in one variable. In order to analyze the interaction of time on pain, fatigue, distress, and interference outcomes and other possible predictive variables, we used generalized estimation equations. All statistics were generated using SPSS version 25 for Windows (IBM, Armonk, NY, USA). Sample size estimation was performed assuming 9 predictor variables, an 80 % power, a 95 % confidence level (0.05), and a 0.2 moderate effect size for Cohen's *f*^2^ for multivariate regression; thus, a sample size of 87 was required.

## 3 Results

**Fig. 3:**
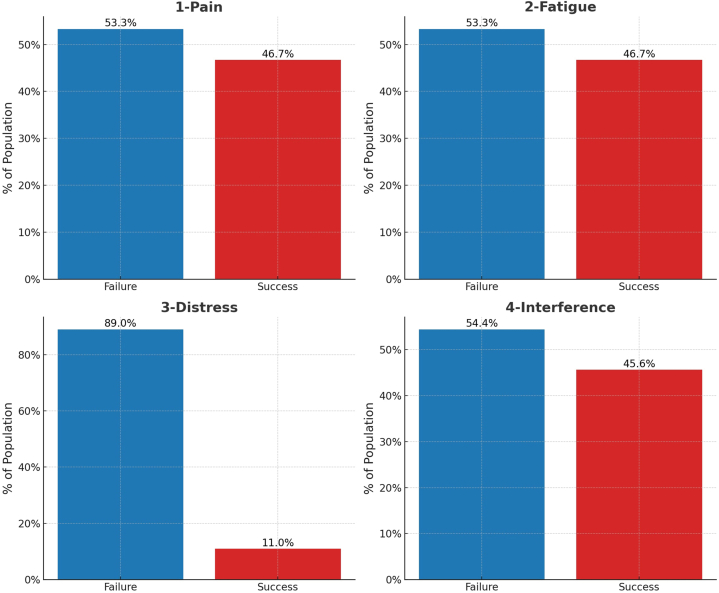
Bar charts showing the percentage of success and failure of the 4 primary study patient relevant outcomes [42,43].

Ninety patients participated in this prospective, multi-center, consecutive case series investigation. The compliance rate for the initial four-week treatment sessions was 95 %, while the compliance for weekly supervised sessions thereafter was 85 %. There were no participant dropouts. However, we had to collect final follow-up data for three patients over the phone due to travel reasons. The median age of the participants was 44 years with an IQR of 38–49, 54.4 % are males, 75.6 % had bachelor's or master's degree, 92.2 % were married. We classified normal BMI as BMI = 18.5–24.9 (66 patients or 73.3 %), overweight patients as BMI = 25–29 (14 patients or 15.5 %), and obese as BMI >30 (10 patients or 11 %); there were no patients with BMI >35 (0 patients) in our sample. Regarding smoking status, 49/90 (54.4 %) are smokers where 12/49 (24 %) patients smoked daily >5 cigarettes per day, 15/49 (20 %) smoked <5 cigarettes per day, 17/49 (35 %) indicated they were intermittent smokers (smoking on a nondaily basis), and 5/49 (10 %) had no available data on smoking frequency. For radicular pain and disc herniation, 70 % had unilateral pain (indicating multi-level disc herniations in these patients), the MSU location in 75.6 % of the participants was AB, however, the MSU magnitude in 92.2 % of the participants was 2. Turning to the radiological exploratory variables, the median of T12-S1 centroid horizontal displacement measures was 7 mm with an IQR 5–10 mm, the median of sacral un-leveling measures was 3° with an IQR of 2–5.38°, the median of lumbo-sacral angle measures was 87° with an IQR 86–89°, and finally the median of lumbo-dorsal measures was 7.5° with an IQR of 5–10°. The 4 study outcomes were pain, fatigue, distress, and interference, the percentage of success in these 4 outcomes were 53.3 %, 53.3 %, 11.1 %, and 45.6 %, respectively. After considering the success in the 4 outcomes together, the main study outcome showed 10 % success (Table 1 and Fig. 3).

**Table 1:** Demographic characteristics of the participants.

Demographic characteristics of the study participants	N = 90^a^
**Age**	44 (38, 49)
**BMI**	
*Normal (BMI* = 18.5 to 24.9)	66 (73.3 %)
*Overweight (BMI = 25–29)*	14 (15.6 %)
*Obese (BMI 30–35)*	10 (11.1 %)
**Gender**	
*Female*	41 (45.6 %)
*Male*	49 (54.4 %)
**Educational level**	
*Bachelor or Master*	68 (75.6 %)
*High school or less*	22 (24.4 %)
**Smoking**	
*No*	41 (45.6 %)
Yes	49 (54.4 %)
Daily >5 cigarettes per day	12/49 (24 %)
Daily <5 cigarettes per day	15/49 (20 %)
Intermittent smokers	17/49 (35 %)
No data on frequency	5/49 (10 %)
Smoking duration	5 ± 3 years
**Marital status**	
*Married*	83 (92.2 %)
*Not married*	7 (7.8 %)
**Type of dominant pain**	
*Bilateral*	27 (30.0 %)
*Unilateral*	63 (70.0 %)
**MSU Location**	
*AB*	68 (75.6 %)
*B or C*	22 (24.4 %)
**MSU Magnitude**	
*3*	7 (7.8 %)
*2*	83 (92.2 %)
**T12 S1 Centroid horizontal displacement**	7 (5, 10)
**Sacral un-leveling**	3.00 (2.00, 5.38)
**Lumbo-Sacral angle**	87 (86, 89)
**Lumbo-dorsal angle**	7.5 (5.0, 10.0)
**Radicular or Leg Pain**	
*Failure*	48 (53.3 %)
*Success*	42 (46.7 %)
**Fatigue**	
*Failure*	48 (53.3 %)
*Success*	42 (46.7 %)
**Distress**	
*Failure*	80 (88.9 %)
*Success*	10 (11.1 %)
**Interference**	
*Failure*	49 (54.4 %)
*Success*	41 (45.6 %)
**Combined outcome (Radicular or leg Pain, Fatigue, Distress, and Interference)**	
*Failure*	81 (90.0 %)
*Success*	9 (10.0 %)

a Median (IQR); n (%).

The results of demographic characters distributed across gender are presented in Table 2. There were no statistically significant differences between males and females in any of the study variables.

**Table 2:** Demographic characters distributed across gender.

Demographic characteristics of the study participants	Gender		p-value^b^
	Female, N = 41^*1*^	Male, N = 49^a^	
**Age**	45 (42, 49)	44 (37, 49)	0.455
**BMI**			0.639
*Normal*	29 (70.7 %)	37 (75.5 %)	
*Obese*	12 (29.3 %)	12 (24.5 %)	
**Educational level**			0.615
*Bachelor or Master*	32 (78.0 %)	36 (73.5 %)	
*High school or less*	9 (22.0 %)	13 (26.5 %)	
**Smoking**			0.773
*Yes*	23 (56.1 %)	26 (53.1 %)	
*No*	18 (43.9 %)	23 (46.9 %)	
**Marital status**			0.121
*Married*	40 (97.6 %)	43 (87.8 %)	
*Not married*	1 (2.4 %)	6 (12.2 %)	
**Type of dominant pain**			0.890
*bilateral*	12 (29.3 %)	15 (30.6 %)	
*unilateral*	29 (70.7 %)	34 (69.4 %)	
**MSU Location**			0.615
*AB*	32 (78.0 %)	36 (73.5 %)	
*B or C*	9 (22.0 %)	13 (26.5 %)	
**MSU Magnitude**			>0.999
*3*	3 (7.3 %)	4 (8.2 %)	
*2*	38 (92.7 %)	45 (91.8 %)	
**T12 S1 Centroid horizontal displacement**	7 (5, 10)	6 (4, 10)	0.418
**Sacral un-leveling**	4.00 (2.00, 5.00)	3.00 (2.00, 5.50)	0.533
**Lumbo Sacral angle**	88.00 (87.00, 89.00)	87.00 (86.00, 89.00)	0.918
**Lumbo dorsal angle**	8.0 (6.0, 10.0)	7.0 (5.0, 10.0)	0.418
**Radicular or Leg Pain**			0.184
*Failure*	25 (61.0 %)	23 (46.9 %)	
*Success*	16 (39.0 %)	26 (53.1 %)	
**Fatigue**			0.184
*Failure*	25 (61.0 %)	23 (46.9 %)	
*Success*	16 (39.0 %)	26 (53.1 %)	
**Distress**			0.750
*Failure*	37 (90.2 %)	43 (87.8 %)	
*Success*	4 (9.8 %)	6 (12.2 %)	
**Interference**			0.255
*Failure*	25 (61.0 %)	24 (49.0 %)	
*Success*	16 (39.0 %)	25 (51.0 %)	
**Combined outcome**			>0.999
*Failure*	37 (90.2 %)	44 (89.8 %)	
*Success*	4 (9.8 %)	5 (10.2 %)	

a Median (IQR); n (%).

b Wilcoxon rank sum test; Fisher's exact test; Pearson's Chi-squared test.

Table 3 shows the outcomes of the medication usage for the 50 patients who continued to use NSAIDs following the initial 6 week course for all 90 participants and the 4 week discontinuation for all patients. Following this 10 weeks for all 90 patients, at the follow-up supervised sessions, NSAIDs were prescribed only to those patients experiencing persistent pain, totaling 50 patients. Out of these 50, 48 patients were reported to have failed to achieve pain relief at the 6-month mark. This observation indicates that medication usage did not improve the odds of success in this subgroup.

**Table 3:** Medication usage in participants and success vs. failure at follow-up.

	Medication status at 6 months		p-value^*2*^
	Receiving N = 50^*1*^	Not receiving N = 40^*1*^	
**Radicular or Leg Pain**			<0.001
*Failure*	48 (61.0 %)	1(46.9 %)	
*Success*	2 (39.0 %)	39 (53.1 %)	
**Fatigue**			<0.001
*Failure*	46 (61.0 %)	2 (46.9 %)	
*Success*	4 (39.0 %)	38 (53.1 %)	
**Distress**			0.002
*Failure*	49 (90.2 %)	31 (87.8 %)	
*Success*	1 (9.8 %)	9 (12.2 %)	
**Interference**			<0.001
*Failure*	45 (61.0 %)	4 (49.0 %)	
*Success*	5 (39.0 %)	36 (51.0 %)	

As shown in Table 4, all the radiological variables were strongly correlated with each other (r > 0.7 and p < 0.001); for this reason, only one of these variables was used in the logistic regression models to prevent the potential multi-collinearity. The lumbosacral angle was the most suitable variable that was selected in all the models as the assumptions of the models were perfectly met by adding this variable. All the potential predictors were put in the models and the assumptions of each model were tested to make sure that each model could fit the data perfectly. Regarding the model showing the predictors of distress, the variables education level, marital status, and MSU magnitude were removed as they showed infinite 95 % CI due to the low sample size (only 10 people from 90 showed success in reducing distress scores).

**Table 4:** Correlation matrix between the radiographic variables.

	T12 S1 Centroid horizontal displacement	Sacral unleveling	Lumbo Sacral angle	Lumbo dorsal angle
T12 S1 Centroid horizontal displacement	1			
Sacral unleveling	0.994 (<0.001)	1		
Lumbosacral angle	−0.796 (<0.001)	−0.791 (<0.001)	1	
Lumbodorsal angle	0.995 (<0.001)	0.993 (<0.001)	−0.794 (<0.001)	1

Computed correlation used spearman-method with listwise-deletion.

As shown in Table 5, the same people who showed success in decreasing pain scores also showed success in decreasing fatigue scores and interference, so the models showing the predictors of pain, fatigue, and interference were the same. Age, education level, and the lumbosacral angle measures significantly affected the odds of success. Increasing age 1 year significantly decreased the odds of success of decreasing pain, fatigue, and interference scores by 15 %, 15 % and 11 %, respectively (pain and fatigue OR = 0.85, p = 0.016; interference OR = 0.89, p = 0.042). High school or lower education significantly increased the odds of success of lowering the scores of pain, fatigue and interference by 26.18, 26.18 and 7.5, the odds when compared with a bachelor's or master's education (p = 0.006, 0.006, and 0.029 respectively). Increasing the lumbosacral angle measures by 1° significantly increased the odds of success of lowering the odds of pain, fatigue, distress, and interference by 3.52, 3.52, 27.99 and 2.55, respectively (p < 0.001, <0.001, = 0.003, = 0.001, respectively).

**Table 5:** Logistic regression models showing the predictors of the 4 outcomes of the study.

	Radicular Pain		Fatigue		Distress		Interference	
Predictors of success	Odds Ratios (95 % CI)	p	Odds Ratios (95 % CI)	p	Odds Ratios (95 % CI)	p	Odds Ratios (95 % CI)	P
Age	0.85 (0.73–0.96)	**0.016**	0.85 (0.73–0.96)	**0.016**	0.88 (0.73–1.03)	0.138	0.89 (0.79–0.99)	**0.042**
BMI [Obese]	0.26 (0.03–1.78)	0.187	0.26 (0.03–1.78)	0.187	0.72 (0.01–26.33)	0.866	0.16 (0.02–0.99)	0.067
Educational [High school or less]	26.18 (3.36–365.34)	**0.006**	26.18 (3.36–365.34)	**0.006**	1.2 (0.98–46.45)	0.8	7.50 (1.40–56.53)	**0.029**
Type of dominant pain [unilateral]	1.33 (0.16–10.72)	0.781	1.33 (0.16–10.72)	0.781	1.35 (0.08–38.89)	0.834	4.12 (0.61–31.65)	0.150
Smoking [No]	1.40 (0.34–6.20)	0.642	1.40 (0.34–6.20)	0.642	0.33 (0.03–2.77)	0.329	1.81 (0.47–7.64)	0.393
Marital status [Not married]	1.54 (0.00–1487.04)	0.943	1.54 (0.00–1487.04)	0.943			2.31 (0.01–909.88)	0.846
MSU Location [B or C]	2.58 (0.41–20.50)	0.325	2.58 (0.41–20.50)	0.325	0.07 (0.00–0.80)	0.067	2.45 (0.44–16.65)	0.320
MSU Magnitude [2]	0.06 (0.00–2.53)	0.157	0.06 (0.00–2.53)	0.157			0.10 (0.00–4.00)	0.266
Lumbo Sacral angle	3.52 (1.94–8.03)	< **0.001**	3.52 (1.94–8.03)	< **0.001**	27.99 (4.99–473.94)	**0.003**	2.55 (1.54–4.91)	**0.001**
Observations	90		90		90		90	

**Fig. 4:**
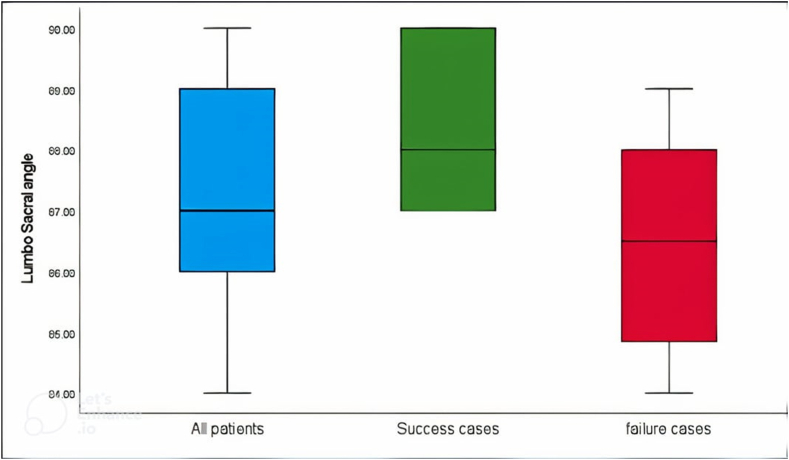
Box plot of the lumbo-sacral angle in all patients, in successful outcome patients and in those with lack of success or failure to respond to conservative care for the combined outcome.

The backward selection method was used to build this model, whereas the variables of education level, marital status, type of pain, and MSU magnitude were removed as they caused infinite confidence intervals due to the small sample size (only 9 people from 90 showed success of the combined outcomes). Both the crude and adjusted odds ratios of the lumbosacral angle measures showed significance, when the lumbosacral angle increases 1°, the odds of success of the combined outcome will significantly increase by 13.24 crude odds and 29.89 adjusted odds (p = 0.002 and 0.003, respectively); shown in Table 6. Fig. 4 depicts the box-plot of the lumbo-sacral angle for: 1) all participants, 2) for patients with success, and 3) for those patients who failed to improve for the total combined outcomes.

**Table 6:** Logistic regression model showing the predictors of the combined main study outcome.

Dependent: Combined Outcome		Crude OR	Adjusted OR
**Age**	–	1.01 (0.92–1.12, p = 0.836)	0.92 (0.76–1.07, p = 0.296)
BMI	Normal	–	–
	Obese	0.32 (0.02–1.86, p = 0.289)	0.85 (0.02–26.16, p = 0.926)
Smoking	Yes	–	–
	No	0.57 (0.11–2.30, p = 0.442)	0.62 (0.06–5.49, p = 0.665)
MSU Location	AB	–	–
	B or C	0.87 (0.12–3.96, p = 0.870)	0.12 (0.01–1.25, p = 0.115)
Lumbo -sacral angle	–	13.24 (3.52–98.70, p = **0.002**)	29.89 (4.95–504.31, p = **0.003**)

As shown in the above GEE model in Table 7, radicular pain scores have significantly decreased by 47 points at discharge and decreased by 38 points after the 6 months of follow-up compared to the scores at baseline (p < 0.001, <0.001, respectively). In our investigation, we did not assess for percentage of canal compromise, cephalad or caudal HNP migration, and type of signal intensity. These variables were not intentionally measured in our study, as we included patients with a percentage (%) of canal stenosis ratio below the cut-off value of 35 % stenosis area. Additionally, the majority of patients (92 %) exhibited a caudal migration pattern of their HNP in the vertical plane. As a result, we did not stratify patients based on HNP migration patterns. However, concerning radiographic variables, when the lumbosacral angle increases 1° (approaching an ideal 90° angle), the pain scores significantly decrease by 4.4 points (p < 0.001).

**Table 7:** Generalized estimation equation table showing the predictors of radicular pain score.

Dependent = Radicular pain scores	Beta	95 % CI^a^	p-value
**Time**			
Baseline	–	–	
After completion of treatment	−47	−51, −44	< **0.001**
After 6 months	−38	−42, −34	< **0.001**
**Age**	0.01	−0.24, 0.25	0.960
**BMI**			
Normal	–	–	
Obese	1.2	−2.6, 4.9	0.540
**Gender**			
Female	–	–	
Male	−1.7	−5.0, 1.6	0.303
**Educational level**			
Bachelor or Master	–	–	
High school or less	3.0	−1.6, 7.6	0.202
**Smoking**			
Yes	–	–	
No	−0.02	−3.2, 3.1	0.991
**Marital status**			
Married	–	–	
Not married	−2.6	−9.0, 3.9	0.437
**Type of dominant radicular pain**			
Bilateral	–	–	
Unilateral	−2.7	−7.3, 1.8	0.237
**MSU Location**			
AB	–	–	
B or C	1.2	−3.4, 5.8	0.614
**MSU Magnitude**			
3	–	–	
2	0.61	−7.6, 8.8	0.884
**Lumbo Sacral angle**	−4.4	−5.7, −3.2	< **0.001**

a CI = Confidence Interval.

As shown in the above GEE model in Table 8, fatigue scores significantly decreased by 48 points at discharge and decreased by 31 points after the 6 months of follow-up compared to the scores at baseline (p < 0.001, <0.001, respectively). When age increased 1 year, fatigue scores significantly increase by 0.38 points (p = 0.012). Those who are graduated from High school or have lower education have significantly decreased fatigue scores by 7.6 points compared to those with a bachelor's or master's degree (p = 0.005). When the lumbosacral angle increases 1°, the fatigue scores significantly decrease by 2.2 points (p < 0.001).

**Table 8:** Generalized estimation equation table showing the predictors of fatigue scores.

Dependent = Fatigue scores	Beta	95 % CI^a^	p-value
**Time**			
Baseline	–	–	
After disposal	−48	−51, −45	< **0.001**
After 6 months	−31	−36, −26	< **0.001**
**Age**	0.38	0.08, 0.67	**0.012**
**BMI**			
Normal	–	–	
Obese	1.8	−2.4, 6.1	0.399
**Gender**			
Female	–	–	
Male	−2.3	−6.0, 1.4	0.232
**Educational level**			
Bachelor or Master	–	–	
High school or less	−7.6	−13, −2.3	**0.005**
**Smoking**			
Yes	–	–	
No	−0.36	−4.0, 3.2	0.842
**Marital status**			
Married	–	–	
Not married	1.5	−4.6, 7.7	0.626
**Type of dominant radicular pain**			
Bilateral	–	–	
Unilateral	1.3	−3.7, 6.3	0.603
**MSU Location**			
AB	–	–	
B or C	0.83	−4.5, 6.1	0.759
**MSU Magnitude**			
3	–	–	
2	7.3	−0.88, 15	0.080
**Lumbo Sacral angle**	−2.2	−3.5, −0.92	< **0.001**

a CI = Confidence Interval.

As shown in Table 9, distress scores significantly decreased by 42 points at discharge and decreased by 19 points after the 6 months of follow-up compared to the scores at baseline (p < 0.001, <0.001, respectively). If age increased 1 year, distress scores significantly increase by 0.39 points (p = 0.013). When the lumbosacral angle increases 1°, the distress scores significantly decrease by 2.5 points (p < 0.001).

**Table 9:** Generalized estimation equation table showing the predictors of distress scores.

Dependent = Distress scores	Beta	95 % CI^a^	p-value
**Time**			
Baseline	–	–	
After disposal	−42	−46, −39	< **0.001**
After 6 months	−19	−24, −14	< **0.001**
**Age**	0.39	0.08, 0.69	**0.013**
**BMI**			
Normal	–	–	
Obese	−1.1	−6.0, 3.7	0.645
**Gender**			
Female	–	–	
Male	−2.0	−5.9, 1.8	0.300
**Educational level**			
Bachelor or Master	–	–	
High school or less	1.1	−3.7, 6.0	0.652
**Smoking**			
Yes	–	–	
No	0.77	−3.0, 4.6	0.690
**Marital status**			
Married	–	–	
Not married	−1.5	−8.1, 5.2	0.667
**Type of dominant radicular pain**			
Bilateral	–	–	
Unilateral	0.04	−5.9, 6.0	0.988
**MSU Location**			
AB	–	–	
B or C	−4.2	−9.7, 1.4	0.142
**MSU Magnitude**			
3	–	–	
2	−4.8	−13, 3.6	0.262
**Lumbosacral angle**	−2.5	−4.0, −1.0	< **0.001**

a CI = Confidence Interval.

Interference scores have significantly decreased by 49 points at discharge and decreased by 28 points after the 6 months of follow-up compared to the scores at baseline (p < 0.001, <0.001, respectively). When the lumbosacral angle increases 1°, the interference scores significantly decrease by 5.1 points (p < 0.001); shown in Table 10.

**Table 10:** Generalized estimation equation table showing the predictors of interference scores.

Dependent = Interference scores	Beta	95 % CI^a^	p-value
**Time**			
Baseline	–	–	
After disposal	−49	−52, −46	< **0.001**
After 6 months	−28	−32, −23	< **0.001**
**Age**	0.24	−0.03, 0.52	0.086
**BMI**			
Normal	–	–	
Obese	3.2	−0.66, 7.2	0.104
**Gender**			
Female	–	–	
Male	−2.2	−5.8, 1.4	0.225
**Educational level**			
Bachelor or Master	–	–	
High school or less	1.6	−3.7, 6.8	0.553
**Smoking**			
Yes	–	–	
No	1.1	−2.5, 4.6	0.554
**Marital status**			
Married	–	–	
Not married	0.27	−6.3, 6.9	0.936
**Type of dominant radicular pain**			
Bilateral	–	–	
Unilateral	−3.8	−8.7, 1.1	0.125
**MSU Location**			
AB	–	–	
B or C	0.20	−4.3, 4.7	0.932
**MSU Magnitude**			
3	–	–	
2	6.0	−2.9, 15	0.185
**Lumbo Sacral angle**	−5.1	−6.4, −3.9	< **0.001**

a CI = Confidence Interval.

## 4 Discussion

The current investigation assessed patients with chronic low back pain and lumbar radiculopathy due to herniation of the nucleus pulposus (HNP). We sought to address if coronal radiological parameters of the lumbo-sacral spine and patient demographic variables would be able to predict the success or lack-there-of in patient relevant outcomes in the PCOQ following a multi-modal physical therapy interventional program combined with relevant pharmacology. Our study's primary hypothesize was that the magnitude of the fractional lumbo-sacral curve (FLSC) or lumbo-sacral angle as used in the current investigation and other AP lumbar radiographic coronal variables could predict the success or failure of treatment in patients with low back pain and a primary complaint of leg or radicular pain due to HNP. We found that the lumbosacral angle statistically impacted the odds of success and failure outcomes on the PCOQ after a course of conservative care and thus, our study's hypothesis is confirmed. In addition to the coronal radiological markers, we found that younger age and lower educational level both impacted the probability a successful outcome.

### 4.1 Demographic breakdown of our population

Understanding the demographic breakdown of our population is important to offer a full context of representation and applicability to the population at large. Our study included 90 participants with lumbar HNP, with a mean age of 44 years (IQR 38–49) and included the following distribution of categories: 1) Gender: 54.4 % male, 45.6 % female; 2) Educational level: 75.6 % had a bachelor's or master's degree, 24.4 % had high school or less education; 3) Marital status: 92.2 % married, 7.8 % not married; 4) Smoking status: 54.4 % smokers, 45.6 % non-smokers; and 4) BMI categories: 73.3 % normal, 15.5 % overweight, 11 % obese. For a comparative analysis, the general population statistics of the United Arab Emirates (UAE) and Egypt, where our study was conducted, indicates the median age in the UAE is approximately 36 years, while in Egypt, it is around 24 years [48]. Thus, our sample's median age of 44 years is higher, reflecting the prevalence of lumbar HNP in middle aged adults [5,8,10]. The gender distribution in the general population is roughly balanced, similar to our study's gender distribution of 54.4 % male and 45.6 % female, indicating representativeness in gender [48]. In the UAE, around 60 % of the population holds a higher education degree, while in Egypt, this figure is lower at approximately 30 %. Our sample's higher percentage of participants with a bachelor's or master's degree (75.6 %) likely reflects the higher likelihood of educated individuals seeking specialized medical care [48].

The general population in the UAE and Egypt has high marriage rates, consistent with our finding that 92.2 % of our participants are married, suggesting that marital status in our sample is representative of the broader population [49]. Smoking prevalence in the UAE is about 25 % among adults, while in Egypt, it is around 24 %. Our participant's higher smoking rate of 54.4 % might indicate a higher prevalence of smoking among individuals with lumbar HNP or a selection bias towards smokers seeking treatment [50]. In contrast, the general prevalence of overweight and obesity in the UAE is 48 % amongst males and 35 % amongst females, and in Egypt, it is around 39.8 %. Thus, our sample has a slightly lower combined prevalence of overweight and obesity at 26.5 % [51]. The above comparison indicates that our study sample is reasonably representative of the general population in terms of gender and marital status [48,49]. However, our sample tends to have a higher education level [48] and smoking rate [50], while it may be accepted if we consider (35 %) are Intermittent smokers. The age distribution aligns with the higher prevalence of lumbar HNP in older adults. Although the prevalence of overweight and obesity is slightly lower, it remains close to the general population's values [51].

### 4.2 Negative predictors: marietal status, smoking and obesity

Patient demographic variables and socio-economics have been found to impact patient outcomes in lumbar disc herniation both conservatively and surgically [[52], [53], [54], [55], [56], [57], [58], [59]]. Our investigation used several patient demographics in an effort to see if they predict the odds of improving the four main scales of the PCOQ (leg pain intensity, fatique, distress, and interferences) after a comprehensive conservative treatment program across 5 similar centers. In contrast to some evidence in the literature [[52], [53], [54], [55], [56], [57]], our results indicate that marital status, smoking habits, and BMI do not alter the odds of recovering. Studies have provided evidence supporting the notion that higher levels of perceived social support and justice correlate with reduced pain severity, interference, and decreased pain-related disability among individuals dealing with chronic pain-related psychosocial conditions but that age, sex, marietal status, and pain duration were not related [52]. Koerner reviewed the literature on the spine patient outcomes research trials (SPORT) and identified that marietal status influenced the 4 year outcomes of patients undergoing surgical discetomy for lumbar herniation, where married patients reported better surgical outcomes [53]. In terms of social support, we only assessed marital status and we did not include an assessment of social injustice and it is not appropriate to compare the findings of conservative treatment to those of surgical [53] interventions.

Specific to lumbar disc herniation, Carlson and Albert [54] updated the SPORT findings and observed that obesity directly impacts the outcomes of surgical intervention, though generally this was only clear for larger BMI's (BMI >35). In the current project, we did not have a large enough sample of high BMI patients (BMI >30) so we used the cutoff of normal BMI <25 and the majority of our population were (73.3 %) classified as normal. Only 14 (15.5 %) of our sample was classified as overweight (BMI = 25–29), and an even smaller number, 10 patients or 11 %, were classifed as obese (BMI >30); there were no patients with BMI >35 in our sample. The lack of a significant number of patients with BMI greater than 30 and no patients with BMI >35 is the likely explanation for our negative fiding herein and the conflict with existing data [54].

In the literature regarding patient smoking status, evidence indicates that smokers tend to have worse overall short and long-term outcomes of both surgical and non-surgical interventions for lumbar disc herniation [[55], [56], [57]]. However, when reviewing the SPORT outcome data, Kerr and colleagues [55] identified that smokers and non-smokers had the same ‘relative’ improvement in outcomes at long-term follow-up, indicating that smokers had greater initial and ending pain and disability levels but the same relative improvement following both conservative and surgical care. Behrend et al. identified that patients that currently smoked had greater initial radicular pain levels and those who continued to smoke during and after intervention had no clinically relevant improvement in pain [56]. In a recent systematic literature review specific to spinal disorders, Rajesh and colleagues [57] identified that smokers experienced a greater need for surgery, greater complication rates, increased pain levels, delayed recovery, and decreased satisfaction after receiving surgery. In the current investigation we identified no association between smoking status and 6-month outcomes on the 4 domains of the PCOQ; this could be due to lack of information on high frequency smoking in our population. Our population of smoking patients contained a significant proportion of individuals with low-rate daily smoking (<5 cigarettes per day) (15/49 patients) and intermittent smoking (smoking on a nondaily basis) (17/49); and these comprised the majority of our smoking population (65 %). Further, the duration of smoking only averaged 5 years in 95 % of our smoking sample. While smoking is not a healthy habit and carries with it risks for increased pain and poor outcomes [57], our results, are not entirely inconsistent with some data in the literature [55,58]. For example, Asher et al. [58] evaluated data from 7547 patients undergoing lumbar surgery for spine disorders and identified that smoking did not affect the 1-year disability index outcomes compared to nonsmokers; but this may be due to younger age and less comorbidities.

### 4.3 Positive predictors: age and education

In terms of positive predictors for the odds of successful improvement following conservative care, our finding that found younger age is a significant predictor of HNP treatment success is consistent with previous investigations [59,60]. In contrast, our study's findings that lower education improves the odds of recovery is in general conflict with previous investigations on this topic [54,[61], [62], [63]]. In the current study, we identified that lower education has significantly increased the odds of success of improving the scores of pain, fatigue, and interference (by 26.18, 26.18, and 7.5, respectively) when compared to patients with a bachelors or masters education. Olson and colleagues reviewed the SPORT HNP cohort and analyzed outcomes based on education levels [63]. Their findings indicate no significant improvement in surgical outcomes across all education levels; but higher education (greater than high-school) was clearly associated with significantly improved conservative care outcomes at four years compared to the lower education groups.

The cause of this discrepancy on education level in our investigation and previous studies [54,[61], [62], [63]] is not fully understood, where possible mechanisms include a direct effect of educational level on occupation; the majority of more educated people in our investigation might be office workers with less physical activity; a potential risk factor for low back pain. Further, we categorized education into only two levels, potentially obscuring heterogeneity that could have been present.

### 4.4 Radiological predictors

A key finding in the current investigation is that the lumbosacral angle significantly affected the odds of success. Problematically, it was identified that the different radiological variables we measured herein, had multicollinearity between them, indicating that these measurements were interrelated. The statistical problem of multicollinearity is that when using multivariate regression modelling these can provide incorrect results even though an individual variable is found to be significant in bivariate analyses. In several of the stepwise regression models, the lumbosacral angle was selected first. Even though we only selected the lumbo-sacral angle, this does not indicate that the other radiologic variables are irrelevant; simply put we omitted the other radiologic variables due to their information being contained by the model that included only the lumbosacral angle.

Recently, investigations have indicated that displacement in the coronal plane negatively affects patient satisfaction and is associated with increased pain, increased loss of function, and decreased quality of life [[25], [26], [27], [28]]. For example, the fractional lumbo-sacral curve (FLSC), defined as the coronal curvature from L4-S1 that is generally in the opposite direction relative to the primary Cobb angle curvature in the lumbar spine, has been found to be a predictor of poor patient outcomes in conservative and surgical interventions in adult patient populations suffering from scoliotic deformities [[29], [30], [31], [32]]. However, we could not locate any investigations looking at the FLSC in chronic low back pain patients with radiculopathy due to disc herniations and thus ours appears to the first conservative trial looking into this as an outcome affecting success of conservative care. The lumbo-sacral angle used in the current investigation is a type of measurement of the FLSC for the distal lumbo-pelvic spine and this measurement was reported two decades earlier [64]. In a population of chronic low back pain patients, Harrison and colleagues [64] reported the reduction of lateral trunk translation following a program of reverse postural training and this trunk translation was shown to improve the lumbo-sacral angles as well as other coronal radiographic measures. It may be that both trunk lateral translation [64] and mild-moderate LLD [[33], [34], [35], [36]] can contribute to increasing the lumbo-sacral angle and that these mechanical spine displacements may be relevant to identify as a predictor of patient responsive to conservative based interventions for this unique population.

The impact of the lumbosacral angle on patient outcomes in our investigation has its origin in a biomechanical explanation. The region between the lumbar and sacral segments is a transition zone at increased risk of injury due to the change in biomechanics where slightly more than 90 % of herniated discs occur at the L4-L5 or the L5-S1 disc space [65]. Eccentric (non-centered) loading, as represented by the coronal lumbosacral angle, can result in asymmetrical stress concentrations in both the pelvis and hip joints [66] as well as the intervertebral disc [67]. While our cases were not true scoliotic deformity patients, it is known that in scoliotic deformities when the trunk shifts toward the convexity of the main curve this puts the patient at increased risk of postoperative coronal imbalance [67]. Thus, spine surgeons have understood that proper correction of the lumbosacral fractional curve is important for successful outcomes for nearly 2-decades [67,68] and we suggest that the lumbosacral angle, herein, is similarly important in LDH cases due to these biomechanical influences.

### 4.5 Optimal treatment frequency, duration, and methods

Treatment of HNP consists of both non-surgical and surgical procedures and there is evidence that many patients prefer conservative treatment over surgery due to a lower risk of complications and lower cost [10]. However, the ideal treatment frequency, duration, and exact multi-modal conservative care for each patient remains obscure. Some investigations have suggested that continued conservative intervention (following a course of 6-weeks) is of minimal value as it increases costs and has limited effectiveness [9]. Surgery is often advised when pain is severe, lasts longer than six weeks, or fails to improve with conservative treatment [11,12]. However, investigations looking at the optimum duration of conservative care and the most effective types treatment for each specific subgroup of patient are still lacking [13]. Thus, the need for investigations identifying patient specific outcomes that predict the odds of successful conservative treatment and the optimum types of interventions are still necessary. Herein, we used a standardized multi-modal conservative care program of medications, education, exercise, physical modalities, and traction. These types of multi-modal care programs are considered evidence based and effective with some disagreement as to the effectiveness of certain interventions existing [[69], [70], [71]]. The recent guidelines from the World Health Organization (WHO) [69] discourage the use of traction in the treatment of pain and disability due to HNP, however, recent information indicates that these WHO recommendations ignore the available data from four separate systematic literature reviews with meta-analysis indicating that traction has good short-term outcomes for radiculopathy associated with HNP [71].

While 4–6 weeks duration seems to be the general consensus, some investigations have shown good outcomes using 8–10 week durations [21,24,71]. The current investigation used a longer duration conservative care program of 10-weeks or more with graded-step-wise interventions that progressed in difficulty and were modified based on the individual needs. Still, a considerable number of patients herein were shown to fail the criteria of a successful outcome on the 4 domains of the PCOQ. First, using only one outcome such as pain may over inflate the odds of patient successes and coupled with a lack of understanding of the minimal important difference for each questionnaire can also alter the odds of success reporting. For these reasons, we used the four different domains and their minimal cut-off values to report success or failure [38,39]. Second, patient specific treatment interventions should be directed at the needs of a given patient and that need should also take into consideration their unique spine deformity. In the sagittal plane, it has been identified that conservative care directed at improving altered sagittal curvature and alignment has increased probability of improving both short and long-term patient outcomes in those suffering radiculopathy [[21], [22], [23], [24],^71^]. It might be possible that correction or reduction of the lumbo-sacral coronal deformity in patients suffering from disco-genic induced lumbar radiculopathy will show positive outcome benefits in a subgroup of patients with this disorder. However, future clinical trials are needed to both test the ability of defined conservative care methods for reducing the coronal radiographic displacements and test the effect of coronal spine correction on both short and long-term patient outcomes.

### 4.6 Limitations and future investigations

Because our investigation was not a randomized controlled trial, it is not possible to identify if the types of treatment we utilized is optimum for improving patients suffering from suffering from chronic lumbar pain and radiculopathy due to HNP. Furthermore, we cannot say whether improving these coronal lumbar alignment variables would result in better success for patients with this condition. We acknowledge that our study categorized education into only two levels, potentially obscuring heterogeneity that could have been present. Further, future investigations need to address variables such as employment, economic resources, health behaviors, and physical and mental health in an effort to identify their impact on patient education and patient outcome predictors as assessed herein. Similarly, due to the lack of patients with BMI >35 and a lack of patients with a high frequency of consistent smoking, we were unable to identify these demographic variables as significant risk factors related to outcomes of patients suffering with pain and disability due to HNP.

Our investigation did not standardize the exact treatment intervention across our 5-centers, however, we intimately chose the 5 centers based on the fact that they used a similar philosophy and treatment approaches to mitigate potential biases. The use of phone interviews to follow up 3/90 (3.3 %) of participants might be considered a limitation, however, the literature supports the reliability and validity of phone surveys [72,73]. Still, we ensured that the phone interviewer was well-trained, followed the same standardized protocol during face to face interviews, and we used the same interviewer for both phone and face-to-face interviews to further maintain consistency and reduce the likelihood of interviewer-related biases. Lastly, we did not stratify patients based on HNP migration patterns, which may indirectly influence treatment outcomes. In our population, we observed a predominant caudal HNP migration pattern in 92 % of patients, indicating a consistent trend in our study population. Also, since this population is from the UAE and Egypt, and also represent a population within a very tight age span (38–49 years), the results from our findings are likely not widely generalizable. Further studies are warranted to explore this aspect in more detail in different geographical areas and with wider age groups.

### 4.7 Conclusions

Our prospective consecutive case series used a multicenter design including 5 similar physiotherapy clinics in the UAE and Egypt. We identified that being younger, less educated, and having more optimal coronal radiological lumbar spine alignment all had a substantial impact on the likelihood of success of 6-month outcomes in patients suffering from chronic lower back pain and radiculopathy due to lumbar disc herniation. Negative (no association) predictors of patient outcomes included marital status, BMI, and smoking and these findings conflict with some existing data in the literature; likely due to our unique sample and categorization of these patients based on specific limits for these variables in our data. Importantly, similar to the fractional lumbo-sacral curve in lumbar scoliotic deformities, the coronal lumbo-sacral angle and lumbar alignment variables (coronal lumbar balance) as reported herein, warrants further investigation as to the effects of this radiographic spine displacement angle on patient relevant outcomes in patients suffering from herniated nucleus pulposus in conservative care outcome investigations. In the future, clinical trials will need to be performed to address correcting coronal plane radiologic displacements of the lumbar spine and its impact on chronic low back pain and radiculopathy due to disc herniation.

## CRediT authorship contribution statement

**Yaser AH. Aljallad:** Writing – review & editing. **Ibrahim M. Moustafa:** Writing – review & editing, Writing – original draft, Supervision, Project administration, Methodology, Formal analysis, Conceptualization. **Mohamed Badr:** Writing – review & editing. **Nouran Hamza:** Writing – review & editing. **Paul A. Oakley:** Writing – review & editing, Writing – original draft. **Deed E. Harrison:** Writing – review & editing, Writing – original draft, Conceptualization.

## Informed consent statement

All participants received informed consent prior to inclusion and participating in this investigation.

## Institutional review board statement

The research was conducted in accordance with international guidelines. The protocol of the study was approved by both the Research Ethics Committee at the University of Sharjah [REC-21-03-11-S] and the Research Ethical Committee at Cairo University [P.T.REC/012/002563].

## Data availability statement

Data will be made available on request.

## Funding

10.13039/100004980CBP Nonprofit (Eagle, ID, USA) approved possible funding of this manuscript for publication fees in the Heliyon. Deed Harrison's role as a senior author and conflicts of interest are outlined below.

## Declaration of competing interest

The authors declare the following financial interests/personal relationships which may be considered as potential competing interests:Paul A. Oakley reports a relationship with CBP NonProfit Inc. that includes: consulting or advisory. Deed E. Harrison reports a relationship with CBP NonProfit Inc. that includes: board membership. DEH teaches rehabilitation methods and is the CEO of a company that distributes products to physicians in the U.S.A. used for the rehabilitation of postural abnormalities. If there are other authors, they declare that they have no known competing financial interests or personal relationships that could have appeared to influence the work reported in this paper.

Deed E. Harrison reports a relationship with Chiropractic Biophysics Seminars that includes: equity or stocks. D.E.H. is the CEO of Chiropractic BioPhysics® (CBP®) Seminars and provides post-graduate education to health care providers and physicians. Spine rehabilitation devices are distributed to physicians through this company. D.E.H. is the president of CBP Non-Profit, Inc., a not-for-profit spine research foundation. If there are other authors, they declare that they have no known competing financial interests or personal relationships that could have appeared to influence the work reported in this paper.
